# The Role of *cis*- and *trans*-Acting RNA Regulatory Elements in Leukemia

**DOI:** 10.3390/cancers12123854

**Published:** 2020-12-20

**Authors:** Irina A. Elcheva, Vladimir S. Spiegelman

**Affiliations:** Division of Pediatric Hematology and Oncology, Department of Pediatrics, Pennsylvania State University College of Medicine, P.O. Box 850, MC H085, 500 University Drive, Hershey, PA 17033-0850, USA

**Keywords:** RNA, leukemia, pediatric leukemia

## Abstract

**Simple Summary:**

Alterations in primary RNA motifs and aberrant expression levels of non-coding RNA molecules have emerged as biomarkers of disease development and progression. Advances in antisense oligonucleotide (ASO) techniques and pharmacologic discoveries in targeting of RNA structures and RNA–protein interactions with small molecules open a new area in RNA therapeutics that may help in developing a next generation of anti-cancer drugs.

**Abstract:**

RNA molecules are a source of phenotypic diversity and an operating system that connects multiple genetic and metabolic processes in the cell. A dysregulated RNA network is a common feature of cancer. Aberrant expression of long non-coding RNA (lncRNA), micro RNA (miRNA), and circular RNA (circRNA) in tumors compared to their normal counterparts, as well as the recurrent mutations in functional regulatory *cis*-acting RNA motifs have emerged as biomarkers of disease development and progression, opening avenues for the design of novel therapeutic approaches. This review looks at the progress, challenges and future prospects of targeting *cis*-acting and *trans*-acting RNA elements for leukemia diagnosis and treatment.

## 1. Introduction

Leukemia, a wide spectrum of blood cancers displaying abnormal proliferation and differentiation capacity of myeloid or lymphoid blood progenitors, is the most frequent type of cancer in children and one of the most common in adults [1]. Acute myeloid leukemia (AML) and acute lymphoblastic or lymphocytic leukemia (ALL) show rapid development and little or no cell differentiation. AML is primarily found in older adults, with a median age of 70 years at diagnosis. Highly heterogeneous clinically and genetically, AML is fatal in about ~80% of elderly patients, and about 60% of people younger than 60 years old [2]. ALL is the most common form of pediatric leukemia, accounting for nearly 30% of all pediatric cancers. While most pediatric patients with ALL achieve remission, 30–35% of these therapies fail, and only 30–40% of adult patients with ALL achieve long-term, disease-free survival [3]. Myelodysplastic syndrome (MDS), myeloproliferative neoplasm (MPN), and chronic forms of myeloid and lymphocytic leukemia (CML, CLL), typically diagnosed in older patients, retain some functional blood cells and develop slowly, but are prone to progression into a hard-to-treat acute leukemia [4,5].

The genetics of adult and pediatric leukemia have been intensively studied [6,7,8,9]. Several studies performed a side-by-side comparison of pediatric and adult myeloid and lymphoblastic leukemia, focusing on protein coding genes with oncogenic and tumor-suppressor functions [10,11]. The research shows that within the same genetic subtypes of ALL, the leukemic cells of older children and adults have more cooperative somatic mutations and a greater enrichment for alterations of epigenetic modifiers than younger patients [12]. The molecular backgrounds of pediatric and adult ALL underlie a profound difference in response to therapies between age categories.

The updated human reference genome, GRCh38.p13, contains 2.95 Gb of sequence, approximately 98% of which does not code for proteins [13]. The primary structure of ~20,000 protein-coding genes is also dominated by non-coding sequences or introns, comprising around 26% of the human genome. Approximately 20% of DNA belongs to structural and regulatory chromosomal sequences, more than 50% of DNA is recognized as intergenic, and only ~2% of DNA is occupied by protein-coding exons. It is believed, however, that at least 80% of the human genome serves a biological purpose beyond defining proteins, either through transcription to functional RNA molecules or other biochemical activities [14,15].

Genes are generally defined as a fragment of chromosomal DNA that is transcribed into a functional RNA molecule or into RNA translated into a functional protein. As such, the total number of human genes is in the range of 40,000 to 50,000 genetic units [16]. However, this is not the whole picture. In addition to a variety of non-coding RNA molecules (ncRNAs) transcribed from intronic, intergenic, and antisense of protein-coding DNA sequences, more than one variant of messenger (mRNA) is usually produced from one gene by the alternative transcription initiation and termination, and the alternative splicing of pre-mRNA. Similar to DNA sequences, the *cis*-acting elements of RNA influence transcripts’ fate internally, while *trans*-acting RNA regulatory factors function through binding with proteins, DNA, or other RNA. Therefore, transcriptomes, a collection of all RNA sequences transcribed from the genomic code, capture gene expression complexity beyond a simple reproduction of a nucleic acid order [17].

Attempts to catalogue genomic variations across the human population brought to light large-scale structural variants in human genomes which were previously disregarded [18,19]. Thousands of deletions, duplications, and copy number variants differ between healthy individuals, suggesting significant variations in transcriptomes. Most of these variants, including individual single-nucleotide variants (SNV) and population-wide single nucleotide polymorphisms (SNPs) are located in non-coding genomic areas and influence gene expression by various mechanisms, e.g., changing promoter or enhancer activity, modifying the primary and secondary structure of non-coding RNA, or altering pre-mRNA processing. Some genetic variants are found to be clinically relevant and can be associated with higher or lower risks of cardio and neurodegenerative diseases [18]. Certain SNPs significantly increase chances of developing cancer [20], including pediatric leukemia [21,22], while acquisition of somatic mutations in non-coding and untranslated regions of RNA transcripts as well as dysregulated expression of ncRNAs acting in *trans* play an important role in neoplasm development and progression [23,24].

Here, we overview the role of *cis*-acting motifs and regulatory ncRNA such as lncRNA, circRNA, and miRNA in blood cancer, giving special attention to pediatric tumors. We discuss how alterations in primary RNA structure and expression levels of regulatory RNA molecules may serve as leukemia biomarkers. Finally, we look at current approaches for chemical and antisense oligonucleotide (ASO) RNA targeting.

## 2. *cis-*Acting RNA Regulatory Motifs

RNA *cis*-acting regulatory motif is a primary RNA sequence, often folded into a distinctive secondary structure (e.g., AU-rich elements (ARE), internal ribosome entry site (IRES)) that regulates fate and activity of RNA itself through interaction with other RNA molecules, DNA, or RNA-binding proteins (RBPs). When located in introns of messenger RNA precursors (pre-mRNA) and untranslated regions (UTRs) of mature mRNA, these regulatory sequences influence pre-mRNA processing, mRNA stability, translation, transport, and decay. The structural integrity of *cis*- and *trans*-acting elements defines the strength and physiological outcomes of their interactions, presenting an additional layer of gene expression control. Alterations in *cis*-acting regulatory RNA motifs are often detected in the context of cancer and other diseases.

### 2.1. Aberrant Pre-mRNA Splicing

Pre-mRNAs are primary transcripts consisting of exons (protein-coding regions with an average size of about 200 base pairs) and introns (much lengthier non-coding sequences flanking exons). Splicing, a process of intron removal and connection of exons by a mega-Daltons spliceosomal complex [25], produces either a constitutive mRNA isoform, or several alternatively spliced mRNA isoforms via differential exon usage. During splicing, the protein components of the spliceosome recognize and bind with *cis*-regulatory pre-mRNA motifs at the 5′ splice site (GT), the polypyrimidine tract (PPT), the 3′ splice site (AG), and the branch point sequence (BPS). Auxiliary or supplemental splice regulatory elements known as exonic and intronic splicing enhancers and silencers are defined by their effects on adjacent splice sites by either promoting or inhibiting exon inclusion [26].

Somatic or hereditary mutations at the exon-intron boundaries and splicing regulatory motifs are the most common types of mutations leading to mRNA mis-splicing. β-thalassemia, a hereditary blood disorder characterized by anemia and a variety of growth and metabolic abnormalities, was one of the first discovered diseases with splicing site mutations negatively affecting production of functional protein (hemoglobin) [27]. Further studies, however, found hundreds of β-thalassemia-causing point mutations in other sites of the β-globin gene and its flanking areas, including promoter regulatory elements, untranslated regions, and protein-coding regions of β-globin mRNA [28].

Relapsed pediatric B-ALL can present with both mutations in protein coding sequences and aberrant splicing of CD19 mRNA. To investigate the molecular nature of resistance to CAR-19 therapies, Thomas-Tikhonenko’s group compared whole exome and RNA sequencing analysis of CART-19 relapse to the CD19-positive, pre-CART-19 leukemia samples from the same patients [29]. Researchers detected hemizygous deletions spanning the CD19 locus, de novo frameshifts, and missense mutations in exon 2 of CD19, which is essential for the integrity of the CART-19 epitope. In addition, researchers identified the alternatively spliced CD19 mRNA variants encoding the intracellular part of CD19, enabling cell-growth promoting stimuli, and a defective extracellular part of the receptor. Authors concluded that both DNA- and RNA-based mechanisms are important for CD19 presentation, and the convergence of acquired mutations and alternative splicing of CD19 enables resistance to CAR-19 immunotherapy. The prominent mutations involving exon 2 skipping and synthesis of truncated cytosolic protein that cannot be targeted by CAR19 were also identified by Fisher et al. [30]. Orlando et al. did not find splice isoforms in a group of 12 patients with relapses after CART-19 and only identified genetic alterations in exons 2, 4, and 5 [31]. Nevertheless, mRNA mis-splicing is a powerful mechanism providing evolutionary advantage to cancer cells and has been documented for adult myeloid [32] and lymphoid leukemia [33].

Genetic studies of leukemia and other types of cancer indicate that splice site mutations span multiple loci in pre-mRNAs and often coincide with aberrations in protein-coding sequences of the gene. This is unlike hereditary disorders caused by a single mutation or changes in several base pairs (e.g., spinal muscular atrophy, myotonic dystrophy) that can be treated with antisense oligonucleotides. In addition, the molecular mechanisms of mis-splicing in cancer are not limited to mutations in pre-mRNA sequences but involve dysfunction of protein and RNA components of the spliceosome [34]. For example, analysis of 2434 whole-genome sequenced donors across 37 tumor types from the Pan-Cancer Analysis of Whole Genomes project identified 277 somatic mutations in U1 spliceosomal small nuclear RNA genes that affected 240 donors across 30 tumor types. Only two positions, base 3 and 28, were mutated in more than 5% of donors in at least 1 tumor type. Mutations, at various frequencies, fall in the stem loop positions and highly conserved 5′ splice-site recognition sequences. The A > C mutation of U1 snRNA was found in 8 out of 78 (10.3%) cases of CLL [35].

Mutations in splicing factor genes are especially common for adult chronic myeloid and lymphoblastic leukemia [36]. Conversely, somatic mutations in splicing factors were not typical for pediatric B-ALL. However, the comparative analysis of splice isoforms in acute pediatric B-ALL lacking mutations in splicing factors genes and normal pro-B-cells identified thousands of aberrant local splice variations per sample [37].

High tissue- or context-specificity is another important characteristic of differential splicing in normal and malignant hematopoietic tissues. The analysis of alternatively expressed isoforms between aging hematopoietic stem cells (HSCs) and progenitor cells (HPCs) identified a significant divergence with only few isoforms of transcription and histone regulators being commonly upregulated [38]. Rojas et al. aimed to identify differentially spliced variants between two hematologic entities with a similar genetic background, 17 p deletion: primary plasma cell leukemia and multiple myeloma. The results of transcriptome analysis reveal a significant deviation between the two types of tumors. Interestingly, most of the differences were observed in the spliceosome machinery genes, which emphasizes the cell type- specificity of alternative splicing [39].

### 2.2. Alterations in Untranslated Regions (UTR) of mRNA

The untranslated regions in mRNA (5′ UTR and 3′ UTR) originate from pre-mRNA exons and flank a protein-coding sequence of mature messenger RNA on both sides of an open reading frame (ORF). The UTRs are rich in *cis*-acting elements and distinctive secondary structures (hairpins) that are recognized by regulatory ncRNA and RBPs. Similar to splicing, recurrent UTRs abnormalities were found in cancer and previously reviewed [23,40,41].

#### 2.2.1. 5′ UTR Alterations in Leukemogenesis

Alterations in 5′ UTRs can disrupt both translation efficiency and protein characteristics. For example, mutations in the 5′ UTR of ANKRD26, the Ankirin Repeat Domain 26 Gene, lead to expression of N-terminally truncated protein and cause the autosomal-dominant form of inherited thrombocytopenia and increase predisposition to AML [42,43]. The rare cases of genetic predisposition to MDS/AML are linked to SNPs in various regions of the *GATA2* gene, including 5′UTR, that cumulatively lead to *GATA2* loss-of-function [44].

With the right sequence context in translation initiation sites (TIS), certain non-AUG start codons can generate expression comparable to a canonical, AUG start codon, whereas mutations in TIS change levels of expression [45]. Endogenous nucleotide repeats expansions upstream of coding-region and a shifts in ORFs is linked to production of abnormal peptides due the repeat-associated non-AUG translation (RAN) common for inherited neurodegenerative diseases [46,47]. A study of 17 patients with the family history of chronic lymphocytic leukemia (CLL) and 32 patients with early-onset B-cell CLL did not observe a pathological CAG repeats expansion [48]. The analysis of polymorphisms in thymidylate synthase 5′-UTR 28 bp tandem repeats found a lower blast counts in ALL patients with 2R2R allele, but no such genotype-dependent differences were observed in AML cases [49].

In the context of stress-related global repression of translation, the production of certain oncogenic proteins can increase due to the stress-induced activation of previously repressed upstream start codons [50]. Sendoel et al. demonstrated that during transformation of skin epithelial cells, certain cancer related mRNAs such as nucleophosmin (*NPM1*) exhibited increased ribosome occupancy in upstream CUG rather than in conventional AUG initiation sites of canonical ORFs. In addition to a selective generation of oncogenic proteins through unconventional start codons, researchers found a shift of transcriptome towards pathways of stemness and mediators of Wnt/β-catenin signaling [51,52]. These findings suggest that the adverse changes in the molecular-genetic profile occur before the early signs of transformation are phenotypically notable.

#### 2.2.2. 3′ UTR Alterations in Leukemogenesis

Alternative cleavage and polyadenylation (APA) are a differential selection of AAUAAA polyadenylation sites in 3ʹUTR by APA factors, leading to the expression of different mRNA isoforms that code for the same protein [53,54]. APA is globally regulated in response to extracellular stimuli that regulate proliferation and differentiation. The first example of 3′UTR shortening was described during T cell activation in response to changes in cell proliferation status [55]. Most fast-proliferating cells, including embryonic stem cells, express transcripts with shorter 3′UTR, though some transcripts, such as those encoding for cell adhesion molecules, may have extended 3′UTR [56,57]. The length of 3′UTR can determine the intracellular protein localization. For example, the long 3′UTR of CD47, a protein conveying antiphagocytosis through the “do not eat me” signal in leukemic cells, enables efficient cell surface expression of CD47, whereas the short 3′ UTR primarily localizes CD47 protein to the endoplasmic reticulum [58].

A meta-data analysis of microarray data by Mayr and Bartel demonstrated that shorter mRNA isoforms in cancer cells display increased stability through the loss of microRNA-mediated repression and typically produce ten-fold more protein [59]. The bioinformatics study of alternative polyadenylation in 358 Pan-Cancer tumor and normal pairs across seven types of cancers identified that 91% of genes expressed in cancer have shorter 3′-untranslated regions (3′ UTRs) to avoid microRNA-mediated repression [60]. A somatic mutation in 3′ UTR, however, can create a new site for miRNAs recognition, causing downregulation of tumor suppressor genes in AML [61].

3′ UTR shortening is associated with increased activity of oncogenes in blood and immune cells. For example, fusion transcripts of the Mixed Lineage Leukemia (*MLL*) gene that lack its native 3′ UTR are associated with the increased activity of those fusions in leukemia cell lines and tumors compared to fusions that retain *MLL* 3′ UTR [62]. Strongly proliferative mantle cell lymphoma (MCL) tumors have exceptionally high *Cyclin D1* mRNA levels, expressing short *Cyclin D1* mRNA isoforms with truncated 3′ UTRs [63].

A study of 452 CLL cases and 54 patients with monoclonal B-lymphocytosis, a precursor disorder, comprised a comprehensive evaluation of recurrent mutations in non-coding regions and found recurrent alterations in the 3′ region of *NOTCH1*, which cause aberrant splicing events, increase NOTCH1 activity, and result in a more aggressive disease [33]. Another study by Lee et al. investigated the oncogenic potential of mRNA processing events in 59 cases of CLL [64]. RNA sequencing revealed the widespread recurrent upregulation of truncated mRNAs and proteins that were caused by intronic polyadenylation. Truncated mRNAs predominantly represented tumor suppressors lacking full-length structure and functionality. Importantly, the role of these genes in cancer was underestimated before due to a lower mutation rate on a DNA level. Therefore, mis-splicing and aberrant polyadenylation can be a driving force of hematopoietic malignancies with few detectible genetic mutations.

Aberrant splicing in 3′ UTR of splicing factor *hnRNPA1* and reduction of its mRNA levels initiate a chain of mis-splicing events affecting oncogenes and tumor suppressors in pediatric B-ALL [37]. This finding suggests that aberrant splicing disturbing 3′ UTRs may be a common mechanism of leukemogenesis for both adult and pediatric patients [65].

## 3. Prospective Therapeutic Value of Targeting Non-Coding Pre-mRNA and mRNA Sequences

Could these genetic alterations disrupting non-coding pre-mRNA regulatory sequences and mRNA UTRs have diagnostic or prognostic value in cancer? A functional analysis of alternative spicing mapping cancer-associated changes to changes in proteins indicates that mis-splicing impacts domains classically affected by somatic mutations in different genes and can be considered as an independent oncogenic process [66]. Therefore, detection of mutations in non-coding sequences disrupting pre-mRNA splicing, mRNA stability, and protein synthesis can have diagnostic or prognostic value. However, data variability should be taken into consideration while exploring alternative and aberrant splicing as a marker of disease development and progression. First, the tissue-specific expression patterns of differentially spliced pre-mRNAs and the adaptive nature of alternative splicing, which changes drastically with microenvironment and age, suggest that genetic analysis of samples with identical genetic background is preferable in order to decrease data inconsistency [67]. Clinically relevant phenotypes such as resistance to therapeutics or tumor repopulating capacity would be the right starting point for identification of splice variants promoting clonal expansion [68]. The standardization of tissue sampling procedures is particularly important for long-term studies, where the occurrence of clonal mutations could change significantly upon treatments [69]. The genetic studies show that cells corresponding to relapse are present in a minor subpopulation at diagnosis [70]. Therefore, technical inability to detect mutations and the rapidity at which mutagenesis occurs may compromise the reliability of genetic testing. For example, the mis-spliced CD19 mRNA isoforms progressing to relapse were detected by Fisher et al. at diagnosis [30]. Another study, however, did not detect the genetic variants found at CART-19 relapse just one month before the disease reoccurred [31].

Although most aberrantly spliced mRNAs undergo nonsense-mediated decay (NMD), the successfully processed and translated messengers can produce atypical, tumor associated neopeptides. As discussed above, alteration in 5′ UTRs of mRNAs can also increase the production of cancer-specific protein isoforms from non-canonical TIS. Hematologic malignances, especially AML, often reveal antigens not expressed by normal cells. That leukemia associated antigens are targeted by αβ and γδ T cells, NKT and NK cells that are proven to be functional against AML in combination with effector ligands and cytokines (perforin, TRAIL, IFN-γ, IFN type I, and IL12) [71,72]. If presented on MHC class I or II of a cell, those neopeptides work as tumor associated antigens (TAAs) and mediate tumor immunogenicity [73]. Seen as foreign by the adaptive immune system, neoepitopes, identified by various approaches, typically associated with better treatment outcomes in solid tumors [74]. Computational analysis of WES from 91 CLLs allowed for prediction of 22 mutated HLA-binding peptides per leukemia. HLA binding was experimentally confirmed for ∼55% of such peptides. Further analysis of WES data on 2488 samples across 13 different cancer types estimated from dozens to thousands of putative neoantigens per tumor, suggesting that neoantigens are frequent in most tumors [75].

The large whole exome sequencing (WES) and RNA-seq studies identified widespread splicing alterations in around 30% of differentially expressed transcripts. Even though many of them are not cancer drivers, those aberrations can contribute to tumor immunogenicity [76]. Jayasinghe et al. bioinformatic analysis indicates that most splicing site-creating mutations (SCMs) were generated within the *TP53* and *GATA3* genes [77]. Tumors with SCMs expressed both T cell markers (PD-1, CD8A, and CD8B) and immune checkpoint blockade PD-L1 molecule, indicating that alternative splice forms induced by SCMs increase the overall immunogenicity of these cancers. The proposition that PD-L1 immunotherapy could be a potential treatment for samples containing SCMs requires further investigation with in vitro and in vivo models of leukemia [77].

## 4. Regulatory Non-Coding RNA Molecules

Several large-scale and single cell sequencing studies explored transcriptomes of normal and malignant hematopoietic cells [78,79,80,81,82]. RNA landscape of the normal human hematopoietic hierarchy, featuring 38,860 unique ncRNAs, 20,466 mRNAs, and 900 miRNAs, displays highly lineage-specific expression of all types of ncRNAs (long non-coding RNA (lncRNA), long intervening ncRNAs (lincRNAs), pseudogenes, antisense transcripts (AS), retained introns, miRNA, and small nucleolar RNAs (snoRNAs)) [79]. The ncRNA expression in leukemia cells is also vastly lineage-specific, often exhibiting pleotropic, context- and concentration-dependent effects on cell physiology. Nevertheless, certain ncRNA loss- or gain-of-function is strongly associated with tumorigenesis and genes encoding those ncRNAs are known as tumor suppressors and oncogenes similar to protein-coding genes [24]. *Trans*-acting ncRNAs regulate gene expression in distal genomic regions while *cis*-acting RNA molecules attenuate gene expression of the locus of their origin or nearby (not to be confused with the internal *cis*-acting RNA motifs discussed above).

Research strategies elucidating the role of ncRNAs in leukemia can be summarized as follows: (i) identification of highly up- or downregulated ncRNA common for certain histological and cytogenetic subtypes of leukemia by analyzing either primary tumors and body fluids, or previously published arrays such as The Cancer Genome Atlas (TCGA) database; (ii) evaluating ncRNAs as potential biomarkers of leukemia in a relationship with white cell blood count, overall survival (OS), event- or disease-free survival (EFS, DFS), minimal residual disease (MRD), and risk of relapse; (iii) mechanistic studies of ncRNA function in a cell through interaction with DNA, RNA, and protein targets. Finally, a large body of work has been dedicated to understanding the role of ncRNA in chemoresistance and developing anti-ncRNA targeted therapies.

### 4.1. Long Non-Coding RNA

Long non-coding RNAs are primary RNA transcripts over 200 nucleotides in length, which are named and categorized based on their genomic origin. Relative to protein-coding sequences, lncRNAs are defined as (i) sense-overlapping, antisense-overlapping, or both (ii) bi-directional, transcribed from sense and anti-sense DNA strands of neighbor genes; (iii) intronic, when transcribed from distal introns; and (iv) intervening/intergenic (lincRNA), not overlapping with annotated coding genes [83,84]. The current version of LncBook lists 270,044 lncRNAs, but only 1867 lncRNA are experimentally validated [85,86]. Long ncRNA expression and processing are similar to protein-coding genes such as promoter conservation and lncRNA splicing. Typically lacking long ORFs, lncRNAs do not produce fully functional proteins. However, lncRNAs with conserved regions comprise three times more ORFs with evidence of translation than non-conserved sequences. In addition, the conserved regions of intergenic lncRNAs, such as *CYRANO, MALAT1, NEAT1* and *MEG3*, are significantly enriched in protein–RNA interaction motifs [85]. The specific, nuclear retention sequences predetermine lncRNA nuclear localization. If those motifs are excluded during splicing, lncRNA can be transported to the cytoplasm [87].

**Figure 1:**
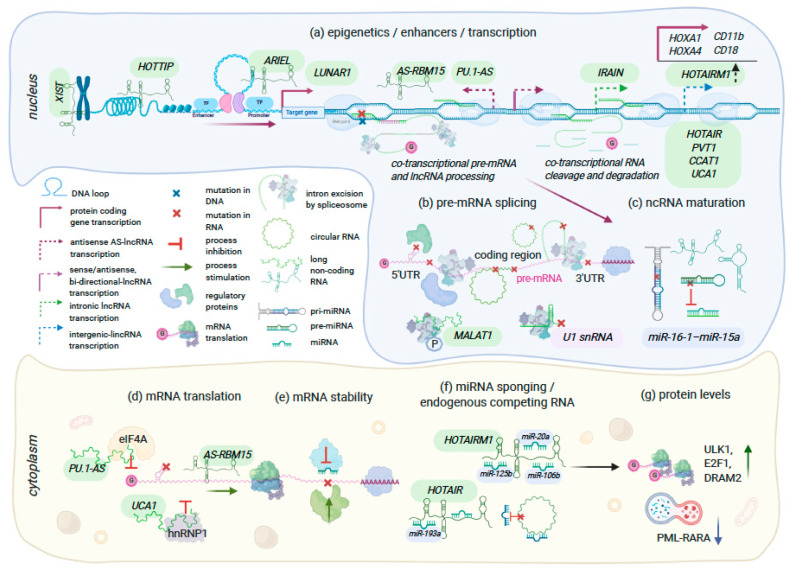
*Cis*- and *trans*-acting RNA regulatory elements, mechanisms of function. *Nucleus* (**a**) Epigenetics, enhancers, transcription: transcribed and processed in the nucleus, most lncRNAs have nuclear localization and involved in chromatin remodeling and transcriptional regulation of gene expression; long intergenic non-coding RNAs (*XIST*, *HOTTIP, ARIEL, LUNAR1* and others) interact with Polycomb complexes and other adapter proteins, form connections between transcriptional regulators and distal DNA sequences through DNA looping; lncRNAs transcribed from antisense to protein-coding genes DNA strands, e.g., *AS-RBM15*, *PU.1-AS*, regulate expression of these genes posttranscriptionally (**d**); both transcriptional and posttranscriptional mechanisms of action were described for some lncRNAs, e.g., *HOTAIR, HOTAIRM1, UCA1*, that regulate gene expression in their genomic locus (acting in *cis*), and distal genomic regions (acting in *trans*). (**b**) pre-mRNA splicing: *cis*-acting pre-mRNA motifs are recognized by *trans*-acting RNA and protein factors during pre-mRNA splicing. Inherited or somatic mutations in splicing regulatory sequences of pre-mRNA cause mRNA mis-splicing; alterations in untranslated 3′ and 5′ areas affect mRNA stability and translation; mutations in the spliceosomal *U1 snRNA* cause global mRNA mis-splicing and aberrant polyadenylation; lncRNA *MALAT1* regulates phosphorylation of splicing factors; circular-RNA are formed through back-splicing of introns. (**c**) ncRNA maturation: germ line mutations in *pri*- and *pre-miRNA-16-1-miR-15a* abolish their maturation. *Cytoplasm* (**d**) mRNA translation: anti-sense lncRNAs (*PU.1-AS* and *AS-RBM15*) regulate expression of protein coding by either promoting or inhibiting translation; *PU.1-AS* and *UCA1* lncRNAs form complexes with translational regulators (eIF4A, hnRNP1) which decreases mRNA translation efficiency; (**e**) mRNA stability: *cis-*acting regulatory elements in 3′ UTR determine mRNA stability; (**f**) miRNA sponging, endogenous competing lncRNA: *HOTAIR* and *HOTAIRM1* sequester specific miRNAs; alterations in endogenous competing RNA influence miRNA levels. (**g**) protein levels: depletion of *miR-20a*, *miR-125b*, and *miR206b* by *HOTAIRM1* increases mRNA stability and translation of autophagy regulators *ULK1*, *E2F1*, and *DRAM2,* and induces PML-RARA degradation.

Through binding with DNA, RNA, and proteins in the nucleus and cytoplasm, lncRNAs influence gene expression epigenetically, co-transcriptionally, and post-transcriptionally, acting as oncogenes [88,89,90,91,92,93,94] or tumor suppressors [95,96,97,98,99,100] in cancer, Figure 1, Table 1.

One of well-studied lncRNAs, *X-inactive specific transcript* (*XIST*) is a large, 17 kb, transcript involved in X-chromosome genes’ inactivation. Several conserved repeats of *XIST* mediate recruitment of the epigenetic Polycomb Repressive Complexes (PRC), initiating gene silencing on X chromosome [106]. Deletion of *Xist* in the murine blood compartment induced highly aggressive MDS/MPN suggesting that *Xist* has a genome-wide impact and acts as a potent suppressor of myeloid blood malignancies [107].

*HOX* gene loci-associated *cis*-acting lncRNAs, *HOX transcript antisense RNA* (*HOTAIR*) and *HOXA transcript at the distal tip* (*HOTTIP*), program active chromatin through interaction with Polycomb and other adapter proteins and play oncogenic roles in leukemia [108,109]. Recently, Luo et al. investigated aberrant activity of *HOTTIP* in AML and showed that *HOTTIP* coordinates *HOXA*-driven topologically associated domain (TAD), including the expression of the posterior HOXA genes. *HOTTIP* also binds in *trans* with promoters of key hematopoietic regulators like *PBX3, MYC, KIT, CD33, MEIS2*, and *RUNX1*. In mice, *Hottip* displayed oncogenic properties leading to AML-like disease by altering the homeotic-hematopoietic gene-associated chromatin signature and transcription programs [91,101].

Oncogenic lncRNA *HOTAIR* sustains leukemia growth and proliferation by negative epigenetic regulation of *p15* genes in the nucleus and by sponging miR-193a away from *c-KIT* mRNA in the cytoplasm [110,111]. Another example of intergenic *trans*-acting lncRNA enhancing oncogene expression through miRNA titration, or a competing endogenous RNA (ceRNA), is *CCAT1.* Often upregulated in M4-M5 subtypes of AML, *CCAT1* inhibits monocytic differentiation and promotes proliferation by reducing *miR-155* availability and consequently increases levels of *c-MYC* [112].

The intergenic lncRNA *HOTAIRM1*, located in *HOXA* cluster, is a tumor suppressor regulating selective induction of *HOXA1*, *HOXA4,* and myeloid markers CD11b, CD18, and CD11c in NB-4 human acute promyeloblastic leukemia [113,114,115]. *HOTAIRM1* expression is associated with myeloid lineage specification and ATRA-driven cell cycle arrest. Another anti-leukemic mechanism of *HOTAIRM1* action implements degradation of PML-RARA oncoprotein and support an autophagy pathway by withdrawing *miR-20a*, *miR-106b*, and *miR-125b* from *ULK1, E2F1,* and *DRAM2* mRNAs [116].

In addition to miRNA sponging, lncRNA are capable of altering protein synthesis by interfering with translational machinery. Daniel Tenen’s group showed that the interplay between *PU.1* sense and antisense RNAs, regulated from shared *cis*-regulatory DNA elements, is important for maintaining physiological dosage of *PU.1* [102]. Originating from an intronic promoter, *PU.1* anti-sense transcript (*PU.1-AS*) disrupts *PU.1* translation between the initiation and elongation steps by selective binding with eIF4A initiation factor [102]. Therefore, elevated expression of *PU.1-AS* leads to downregulation of PU.1 and promotes myeloid leukemia [103]. Conversely, *AS-RBM15,* an anti-sense RNA transcribed in the opposite direction within exon 1 of the megakaryocytic regulator RBM15, promotes terminal differentiation of hematopoietic progenitors by enhancing RBM15 translation in a 5′ cap-dependent manner. The overlapping region between *AS-RBM15* RNA and 5′ UTR of *RBM15* mRNA functions as an enhancer of RBM15 protein synthesis in megakaryocytic leukemia [95].

To evaluate the prognostic significance of differentially expressed lncRNA in the genetically diverse AML, the de novo RNA sequenced bone marrow samples or TCGA data were thoroughly investigated by several groups. Garzon et al. identified a small subset of lncRNAs strongly correlated with the treatment response and survival of elderly patients (>60-year-old) with cytogenetically normal, untreated AML harboring *FLT3-ITD, NPM1, CEBPA, IGD2*, and *RUNX1* mutations [117]. The follow-on study of cytogenetically normal acute myeloid leukemia in younger adults (<60 years old) identified 24 lncRNAs associated with event-free survival. Interestingly, among genetic aberrations with prognostic values only tumors with *NPM1, CEBP*A, and *FLT3-ITD* mutations displayed differential lncRNA expression [118]. A novel prognostic marker, lncRNA *XLOC_109948,* was identified by Etienne De Clara et al. in the large-scale bioinformatic analysis of *NPM1*-mutated AML [119]. Low expression levels of *XLOC_109948* were associated with good treatment outcomes. Downregulation of *XLOC_109948* in a *NPM1*-mutated OCI-AML3 cell line treated with Ara-C or ATRA enhanced apoptosis, thus suggesting the role of this lncRNA in drug sensitivity [119].

lncRNAs contribute to proliferation [104,120,121,122,123,124,125,126], chemoresistance [105,127], and shorter overall survival [128,129,130,131,132] in childhood leukemia, while functions of some highly upregulated and downregulated lncRNAs are still unknown [133], Table 2. Urothelial carcinoma-associated 1 (*UCA1*) lncRNA was upregulated in some pediatric AML after adriamycin (ADR)-based chemotherapy [105]. Knockdown of *UCA1* increased the cytotoxic effect of ADR and inhibited HIF-1α-dependent glycolysis in ADR-resistant AML. Mechanistically, *UCA1* positively regulates hexokinase 2 and HIF-1α-dependent glycolysis in HL-60 APL by sponging *miRNA-125a.* In the ADR resistant T-ALL, antisense *CDKN2B-AS1* positively regulates TRAF5 by sponging miR-335-3p [127].

The molecular analysis of different subsets of pediatric B-ALL (t (12; 21), *TEL-AML1*; t (1;19) *E2A-PBX1*; and t (4;11) *MLL-AF4*) identified most differentially regulated lncRNAs (*BALR- 1*, *BALR-2*, *BALR-6*, and *LINC00958*) [134]. High expression levels of B-ALL associated long RNA-2 (*BALR-2*) and B-ALL associated long RNA-6 (*BALR-6*) are linked with shorter overall survival, while their inhibition decreases cell proliferation and induces apoptosis [134,135]. The mechanistic studies of their oncogenic properties in human and mouse B-ALL cells indicate that *BALR-2* inhibits the glucocorticoid receptor signaling pathway, while *BALR-6* negatively regulates activity of *SP1(PU.1*) and its downstream targets.

Subtype-specific ncRNA, including lncRNA, in the six major subgroups of pediatric AML (i.e., inv(16), t (8;21), t (10;11), t (9;11), acute megakaryoblastic leukemia (AMKL), and Down syndrome myeloid leukemia (ML-DS)) were described by Schwarzer et al. [79]. The researchers identified ncRNA stem cell signature which includes HSC-related ncRNA transcripts and ncRNA associated with differentiation. Interestingly, transformed AML blasts expressed a conserved HSC program independent from differentiation-associated ncRNAs, similar to protein-coding genes [136,137]. The downregulation of the differentiation-associated ncRNAs, but not expression of HSCs-associated ncRNAs, was associated with statistically significant poor prognosis [79].

A comprehensive genomic study of 5037 tumor samples and 935 cancer cell lines among 13 types of cancer, including leukemia, revealed both commonly expressed and cancer-type specific lncRNAs [138]. Compared to corresponding normal tissues, 15% of significantly upregulated and 11% of downregulated lncRNA were detected in several cancer types, with *PCAT7, PVT1,* and *HOTAIR* among the most commonly expressed lncRNAs. The somatic copy number alterations (SCNA) via SNP microarray showed that ovarian and lung cancers had the most of high-frequency (>25%) loss- or gain-of-function lncRNA SCNAs. Whereas AML displayed very few SCNAs, high expression of *Breast Cancer Associated lncRNA8* (*BCAL8*) correlated with poor prognosis. Cancer-associated index SNPs were located in 11.7% of lncRNA loci, and roughly half of them were found in close proximity to protein-coding genes.

Gao et al. analyzed the impact of somatic mutations and lncRNA expression across 17 cancer types, and its connection with miRNA expression, methylation, and TF-lncRNA interaction [139]. The scientists found that lncRNA genes located on chromosomes 17 and 1 are more frequently involved in cancer, about 54% of lncRNA mutations occurred only in one cancer type, and only 0.27% were dysregulated in more than eight cancers allowing them to be classified as “common” for the given cohort. Importantly, most of those lncRNAs function as regulators of chromatin assembly and transcription and have a cancer biomarker potential for prediction of susceptibility to cancer, association with disease recurrence, and poor survival rates [140].

### 4.2. Circular RNA

Circular RNAs (circRNAs) are single-stranded RNA sequences covalently linked into circles that range from 100 nt to over 4 kb in size. Similar to miRNA and lncRNA, they comprise evolutionary conserved genomic regions. The biogenesis of circRNAs is linked to splicing and circularization, so-called back-splicing, of exonic, intronic, and other non-coding fragments of newly transcribed RNA. Similar to lncRNA, circRNAs can be translated to proteins and negatively regulate miRNA function by competing with their RNA targets.

The stable structure of circRNAs suggests a long-lasting effect on cellular physiology, making circRNAs suitable diagnostic and prognostic markers. In fact, circRNAs, most intensively studied in AML, were identified as potential biomarkers that can be applied at diagnosis, remission, or associated with resistance to therapy [141].

For example, circ-RNA microarray screening of 115 human samples revealed a strong association of *hsa_circ_0004277* expression with AML development: *hsa_circ_0004277* levels were significantly downregulated at diagnosis and normalized in remission [142]. *circ-ANAPC7* was also proposed to be an additional marker to identify AML [143], but further studies with a larger number of AML samples and normal progenitor controls are required to confirm these observations. The analysis of 113 AML patients and 42 healthy donors identified that circular RNA originating from the *Vimentin* gene (*circ-VIM*) is significantly upregulated and associated with shorter survival in patients with non-acute promyelocytic leukemia and cytogenetically normal AML [144]. High levels of Vimentin itself, a type III intermediate filament that maintains cell integrity, is also associated with AML aggressiveness (e.g., higher count of white blood cells and low overall survival), especially in older patients [145]. Though not capable of carcinogenic transformation on their own, f-circular-RNA produced from fusion genes can promote leukemia development and resistance to therapies [146]. *circPAN3* was shown to contribute to drug resistance through the *circPAN3-miR-153-5p/miR-183-5p-XIAP* axis [147]. Another promising circ-RNA marker is *circ-PVT1*. Similar to *lncRNA-PVT1*, one of the most common long non-coding RNAs, *circ-PVT1* is upregulated in AML and ALL and promotes cell proliferation through supporting c-MYC expression by sponging *let-7* family and *miR-125* [148,149].

Analysis of 12 pediatric AML and healthy controls identified 273 upregulated and 296 downregulated circRNAs, mostly transcribed from chromosomes 1, 6, and 16. Among 20 highly upregulated circRNAs, *circ-0004136* acts as a sponge for several pediatric AML-related miRNAs. The bioinformatic algorithm indicated that target genes involved in the *circ0004136-miRNA-mRNA* network are enriched in leukemia-related signaling pathways. *Circ-0004136* expression was significantly upregulated in pediatric AML and potentially sponged AML-related miRNAs, such as *miR-29a* and *miR-142* [150]. Studies of pediatric B-ALL revealed upregulation of circRNAs associated with *MLL* fusion partner *AF4, circAF4,* and other oncogenes (*AF6, AF9, AF10, ENL, GAS7, PAX5*, *PVT1,* and *HIPK3*) [151,152].

*Cis*-acting RNA motifs determine biogenesis and functions of circRNAs [153]. Typically referred to as the repetitive and non-repetitive long flanking introns of pre-mRNA, altered *cis*-acting elements can potentially abolish or increase expression of circRNAs. The genome-wide in silico search for genetic variants of human circRNAs and analysis of cancer datasets showed that chromosome 17 has a relatively large number of health-related genetic circRNA variants, chromosome 7 contains the highest number of complex mutations, and chromosomes 2 and 1 exhibited the highest number of cancer-related variants. The circRNA-related genetic SNPs, insertions and deletions (INDEL) that might be common for multiple circRNAs have not yet been reported [154].

### 4.3. Short Non-Coding RNAs

The small and medium size, 18-200 nt, non-coding RNAs, e.g., small interfering RNAs (siRNAs), micro RNAs (miRNAs), PIWI-interacting RNAs (piRNAs), small nuclear RNA(snRNA), small nucleolar RNA (snoRNA), promoter-associated small RNAs (PASRs), transcription initiation RNAs (tiRNAs), telomere small RNAs (tel-sRNAs), centrosome-associated RNAs (crasiRNAs), and many others, compose an array of endogenous molecules regulating multiple processes in a cell at the transcriptional, co-transcriptional and posttranscriptional levels. Among all classes of short ncRNA identified to date, miRNAs role in cancer has been investigated most thoroughly [155].

#### miRNA

Single or clustered genes encoding primary miRNA transcripts (pri-miRNAs) ultimately processed into short, ~22 nucleotide sequences, are dispersed throughout the genome and mostly conserved among species. Transcribed by RNA Polymerase II, pri-miRNAs undergo processing by Drosha complex in the nucleus. The processed long miRNA precursors (pre-miRNAs) are exported to the cytoplasm by exportin 5 and cleaved into double-stranded short precursors of miRNAs. After a double-stranded miRNA is loaded into RISC complex, one of the RNA strands, the passenger, is removed, allowing the seed sequence of miRNA to pair with mRNA targets. The main characteristic of miRNA gene silencing pathways is that the single-stranded miRNAs facilitate translational repression and mRNA destabilization through imperfect base-pairing typically with the 3′UTRs.

The first evidence of miRNA gene dysfunction provoking a blood malignancy was reported in 2002 by Calin et al. [156]. The polycistronic RNA encoding for the precursor of *miR-15a-1* and miR-*16b-1* was missing in 70% of B-cell chronic lymphoblastic leukemia with translocation at 13q14. Several powerful oncogenes promoting CLL such as *Cyclin D1*, *MCL1*, and anti-apoptotic factor *BCL2,* were identified as the downstream targets of *miR-15a-1* and miR-*16b-1* [157].

Another vivid example of tumor suppressor miRNAs are *miR-145* and *miR-146a*, which are dysregulated in 5-q syndrome, a subtype of MDS characterized by severe anemia, variable neutropenia, and atypical megakaryocytes. The deletion of 1.5 Mb DNA on the long arm of chromosome 5 (del5q) leads to *miR-145* and *miR-146a* loss-of-function and a subsequent upregulation of Toll–interleukin-1 receptor domain–containing adaptor protein (TIRAP) and tumor necrosis factor receptor–associated factor-6 (TRAF6), triggering phenotypical and functional features of MDS [158].

The amplification of genomic loci *C13orf25* (*MIR17HG* gene, at 13q31-q32) encoding the *miR-17-92* cluster was found in diffuse large B-cell lymphoma, the cancer of immune cells residing in the lymph nodes. Also known as oncomiR-1, the well-studied miR-17-92 cluster consists of six miRNAs (*miR-17, miR-18a, miR-19a, miR-20a, miR-19b-1,* and *miR-92a-1*) important for cell cycle, proliferation, apoptosis, and other pivotal processes. The *miR-17-92* cluster is often dysregulated in hematopoietic and solid cancers. Transactivated by *c-MYC, N-NYC, MXI1,* and other transcription factors, miR-17-92 cluster increases cell proliferation and survival by inhibiting several critical tumor suppressors such as *PTEN* and pro-apoptotic factor *p21* [159].

Another miRNA family playing an essential role in AML, CLL, and lymphomas is *miR-29* (isoforms *miR-29a, miR-29b*, and *miR-29c*). However, *miR-29*s role in blood and other malignancies is dual as they can act as oncogenes or tumor-suppressors in different histological types of tumors [160]. The context- and dose-dependent roles were reported for several miRNAs in various cancers [24,161]. For example, *miR-125b* overexpression is shown to induce either myeloid or lymphoid leukemia depending on the time course and expression levels of *miR-125b* [162,163]. Narayan et al. demonstrated that forced expression of *miR-155* to high levels (>50-fold above controls) displayed antitumor activity in different types of AML (*MLL-AF9*, *MLL-ENL*, and *HoxA9/Meis1*). Conversely, moderate upregulation of *miR-155* was associated with alternative target selection, repression of myeloid differentiation genes, and with leukemic phenotypes in vitro and in vivo [164]. *MiR-126* regulates quiescence and self-renewal in normal and malignant human hematopoietic stem cells with distinct outcomes [165,166]. Surprisingly, both overexpression and knockout of *miR-126* promote leukemogenesis in *AE9a*-induced mouse model [167].

Aberrant expression of miRNA in various subtypes of myeloid and lymphoid leukemia was extensively investigated, and thoroughly reviewed [168,169,170,171]. In addition, miRNAs detected in body liquids and peripheral blood mononuclear cells from adult and pediatric leukemia patients were evaluated as biomarkers. For example, low levels of tumor suppressor miR-206 in serum of pediatric AML patients were associated with upregulated *Cyclin D1* and unfavorable prognosis [172]. By examining miRNA expression in normal blood cells, de novo and relapsed pediatric ALL, Rzepiel et al. found that miR-128-3p and miR-222-3p expression correlates with minimum residual disease (MRD). However, the routine methods of MDR detection were more sensitive and technically reliable [173]. Since miR-150 was identified as one of the most abundant miRNAs in chronic lymphoblastic leukemia, several studies reported both high and low miR-150 levels correlating with poor clinical outcomes in CLL patients. Interestingly, cellular and serum levels of miR-150 were associated with opposite clinical prognoses: low cellular and high serum miR-150 levels were associated with the disease burden [174], indicating that some other cells could possibly be releasing miR-150. The discrepancies between studies evaluating circulating miRNAs can be explained by tissue specificity e.g., serum, plasma, or other body liquids may contain different levels of the same miRNA, and normalization methods used in quantitative polymerase chain reaction analysis.

Similar to other classes of ncRNA, abnormal miRNA expression and processing in cancer are caused by structural and functional changes in the human genome: chromosomal rearrangements, deletions, amplifications, and deregulated epigenetic and transcriptional control of gene expression. Although copy number alterations (CNAs), amplification, and deletions are powerful genetic mechanisms of miRNA deregulation, they are not quite common for AML. By studying 113 cases of AML, Ramsingh et al. show that only 18% of patients have CNAs involving miRNA genes, while multiple alterations in epigenetic and transcriptional regulators are in charge of miRNA abnormal expression [175].

Germline variants in miRNA genes may have a profound effect on miRNA transcription and maturation [176,177]; however, there are lower numbers of SNPs in miRNA genes than in other regions of the human genome, and the polymorphisms mostly affect the regulatory pri-miRNA and pre-miRNA sequences rather than seed motifs [178,179,180]. Sequencing analysis of miRNAs that are dysregulated in CLL identified mutations in the primary precursor of *miR-16-1–miR-15a* that alter the processing of these miRNAs and can cause loss of function similar to a deletion [181]. Accordingly, somatic mutations within miRNA seed regions are rare genetic events [182,183].

## 5. Therapeutic Approaches for Targeting RNA Molecules

Traditionally, therapeutic approaches for targeting a primary RNA structures were based on introduction of complementary DNA or RNA oligonucleotides, or their chemical equivalents, into the target cells. Oligonucleotides can function through RNase H-mediated RNA degradation, RNA interference (RNAi), or through a non-degradative steric hindrance mechanism by replacing or repressing RNA-binding proteins [184].

Meant to silence gene expression by inducing degradation of target mRNAs, double-stranded siRNAs and single-stranded antisense oligonucleotides (ASOs/AONss) are designed to perfectly match the target sequence. Synthetic miRNAs are introduced into a cell either to replace downregulated endogenous miRNAs (RNA mimics) or block the endogenous miRNAs, which resembles an antisense approach. Dorrance et al. demonstrated a successful *miR-126* targeting by the transferrin or anti-CD45.2 antibody-conjugated nanoparticles containing antagomiR-126 both in vitro, in CD34+ blasts sorted from primary elderly AML patients, and in vivo, using *Mll* PTD *Flt3* ITD mouse model [185]. AntagomiR-126 treatments led to ~80% decrease in miR-126 levels in CD34+ blasts and were accompanied with a significant reduction of long-term colony forming cells frequency and a depletion of quiescent CD34+ subfraction as examined by serial replating assays [185]. While multiple preclinical studies showed therapeutic potential of miRNA mimics and antagomirs in leukemia cell cultures and animal models, none of them seemed to move forward with the clinical trials [169]. The first miRNA mimic to treat solid tumors, MRX34, entered the clinic in 2013 [186]. MRX34 was designed to restore expression of vastly downregulated *miR-34a*, which directly regulates at least 24 known oncogenes. At some point, the trial was stopped due to life-threatening immune responses in several patients, but, ultimately, the study was competed using dexamethasone premedication and dose-escalation protocols. Overall, MRX34 demonstrated an acceptable safety for most of the patients and showed the evidence of antitumor activity in a subset of patients with refractory tumors [187].

The 18 clinical trials of anti-sense therapies in chronic and acute leukemias targeted exclusively transcriptional regulators, mostly *BCR-ABL*. While some studies reported a significant improvement in survival for particular groups of patients [188], AONs stability, the targeted delivery to tissues, immunogenicity, and off-target effect remain major obstacles for oligonucleotide-mediated therapies [189]. The proof of concept studies using structurally stable, resistant to nucleases double-stranded LNA GapmeRs, e.g., against *lnc-THADA4-1* in Juvenile Myelomonocytic Leukemia (JMML) [132], and antisense double-stranded DNA oligonucleotides (ADO) against *BCR-ABL* in CML [190], suggest that RNase H-mediated RNA degradation is a potentially effective therapeutic strategy, that requires further validation in vivo.

Delivery efficiency remains one of the important problems in nucleotide-based therapies. Therapeutic molecules can be trapped in endosomes, lysosome or disposed through exocytosis and, therefore, remain inactive [191,192]. Delivering RNA therapeutics to the specific cell types is another challenge. Most of the delivery technologies, including advanced, non-immunogenic lipid nanoparticles (LNPs) loaded with modified RNA, cannot distinguish between various cell types causing off-target effect and reducing desirable outcomes. Dan Peer’s group developed a modular platform for targeted RNAi therapeutics named ASSET (Anchored Secondary scFv Enabling Targeting), which coats the LNPs with monoclonal antibodies [193]. Recently, Veiga et al. utilized ASSET platform and mRNA loaded LNPs for targeted gene expression in Ly6c+ inflammatory leukocytes, and achieved a selective protein expression in vivo [194].

Several commercially viable AON-based therapies are currently FDA approved, and are aimed to treat cytomegalovirus (CMV) retinitis, common in people with a compromised immune system, and hereditary conditions such as Duchenne muscular dystrophy (DMD) and spinal muscular atrophy (SMA) [195]. In the inherited degenerative diseases, AON-based therapies demonstrate partial or full restoration of protein functions by modulating the altered splicing and translation [196].

A combined high-throughput screening of antisense oligonucleotides and small molecules identified compounds promoting exon 51 skipping in dystrophin pre-mRNA [197]. Similar screens identified small molecules inducing desirable splicing phenotype for SMA and enhancement of the survival motor neuron (SMN) protein levels, improving motor functions in mice [198]. Interestingly, RNA-seq analysis indicated that compounds were quite selective and did not have a widespread effect on the transcriptome. This discovery opened a new perspective in targeting of RNA primary and secondary structures by chemical compounds as well as inhibiting RNA–protein interactions in human disease [199]. Prior to this, the interaction of small molecules with RNA were extensively studied in viruses. For example, small molecules were shown to interfere with the HIV transactivation response and Rev response element [200].

Velagapudi et al. investigated oncogenic non-coding RNA targeting by known anti-cancer drugs [201]. The team described a small molecular microarray-based approach, AbsorbArray, which allows for unmodified compounds, including FDA approved chemotherapeutics, to be probed for binding to RNA motif libraries in a high-throughput format. The primary screening identified that topoisomerase inhibitors bind the Dicer site of *pre-miR-21* and inhibit *miR-21* biogenesis. In vitro, these compounds, e.g., mitoxantrone, reduced mature *miR-21* levels and modulated *miR-21*-mediated invasive phenotype. Importantly, the chemical crosslinking and a pull-down assay (Chem-CLIP) studies confirmed physical interaction between *pre-miR-21* and the small molecule. Among different classes of compounds, topoisomerase inhibitors, kinase inhibitors, and splicing modulators were key classes that bound RNA [201].

The high-throughput methods for investigating chemical compounds targeting RNA molecules and mechanisms of drugs targeting RNA–protein interactions were recently reviewed. Anita Donlic and Amanda Hargrove placed a unique emphasis on the specifics of RNA structural elements or RNA-mediated interactions that enable disease-related functions in mammalian systems as well as the phenotypic changes observed upon treatment with targeted ligands [202]. Zhu et al. provide a comprehensive overview of the commercialized RNA-mediated therapies and those that are under clinical investigation [203]. A recent review by Peng Wu discusses the selective strategies for targeting RNA-binding proteins, and the high-throughput screening approaches to identify inhibitors of RNA–protein interactions [204]. A common theme of these and similar articles highlights the importance of understanding the principles of RNA-ligands efficient design and producing libraries of more specific RNA-binding chemotypes. For more effective pre-clinical assessment, RNA and RBP inhibitor testing systems should include cellular assays investigating interactions and metabolism of full-length molecules in a cell and animal models.

## 6. Concluding Remarks

Once defined as architects of eukaryotic complexity and the dark matter of cancer genomes [23,205], ncRNA molecules could represent important yet challenging therapeutic targets due to their pleotropic and context-dependent effect. The dual role of posttranscriptional regulators acting as oncogenes and tumor suppressors, however, is not limited to RNA molecules, but RBPs as well. Therefore, understanding RNA metabolism in living systems and selecting ribonucleoprotein targets that are best suited for therapies is as important as understanding their structural characteristics.

Another level of RNA network complexity lays in the abundance and variety of ncRNA interactions with mRNA and other ncRNA molecules. The multifaceted ncRNAs acting as transcriptional, co-, and posttranscriptional regulators indicate the importance of understanding the circuitous architecture of the RNA network. Although the selective targeting of upregulated oncogenic RNA molecules may seem a step towards personalized medicine, in most clinical settings only a limited number of patients respond to targeted therapies that address a single genetic abnormality [2]. Thus, targeting key elements of regulatory modules or common structural elements affecting multiple targets could be a more effective strategy against genetically heterogeneous blood cancers.

Understanding the functional significance of the somatic point mutations and genomic variants located in non-coding and untranslated regions of the genome is also a challenge since they can influence the expression of distal genes at both transcriptional and posttranscriptional levels. Annotation of twenty-three million regulatory SNPs that are involved in a wide range of processes, including proximal and distal transcriptional and posttranscriptional regulation of gene expression, indicates that roughly half of them are involved in RBP- and miRNA-mediated posttranscriptional regulation [206]. A global high-resolution search for protein RNA-binding domains led to the observation that mutations causing monogenic diseases, ~10,000 human diseases including sickle-cell anemia, were enriched in genomic regions encoding for unconventional RNA–protein interactions [207]. Therefore, the role of *cis*- and *tran*s-acting RNA regulatory elements and RBPs in human disease might be larger than currently known.

The concept of RNA-targeting therapeutics using ASO, siRNA, miRNA and other synthetic RNA has been proven to be effective in some degenerative diseases. The efficient and safe targeted delivery of RNA therapeutics into specific tissues will be key for expanding those approaches to other clinical indications including cancer. Recent discoveries in the chemical targeting of RNA motifs and identification of small molecules disrupting RNA–protein and RNA–RNA interactions open a new area in RNA therapeutics that may help in developing a next generation of anti-cancer drugs.

## Figures and Tables

**Table 1:** Examples of nuclear and cytoplasmic function of long non-coding RNAs.

lncRNA Gene Name	Type of Cancer	Expression in Cancer	Mechanisms	Gene Expression Regulators	Ref
**Nuclear Function: Chromatin Folding and Transcription**					
*HOTTIP*	*MLLr^+^ NPM1^C+^* AML	Upregulated	Remodels chromatin accessibility and alters hematopoietic transcription programs affecting multiple pathways (cell cycle, apoptosis, myeloid/leukocyte cell differentiation, JAK-STAT signaling, and regulation of cell development); promotes HSCs self-renewal leading to AML-like disease in mice; lower survival in AML patients	CCCTC-binding factor (CTCF) active at a binding site located between *HOXA7* and *HOXA9* genes (CBS7/9); Psip1/p52	[91,94,101]
*MAGI2-AS3*	AML	Downregulated	Inhibits self-renewal in leukemic stem cells by promoting *TET2*-dependent DNA demethylation of the *LRIG1* promoter in acute myeloid leukemia; a better survival with overexpression	Unknown	[98]
*IRAIN*	AML	Downregulated	Intrachromosomal interactions, enhancer-promoter loop within *IGF1R* gene	Unknown	[100]
*MALAT1*	AML, CLL, CMML, MM, HCC, other cancers	Upregulated	Regulates the phosphorylation status of serin-rich splicing factors (SRSF), their subcellular localization in HeLa cells; interacts with PCR2, transcription factors and sequesters miRNA in the cytoplasm. Aberrant expression in Del 13q14 CLL; *MALAT1* depletion increases cytarabine sensitivity in AML and response to ATRA-treatment in CMML	Multiple transcription factors e.g., SP1, SP3, HIF1-alpha, c-MYC	Reviewed in [93]
*CASC15*	*RUNX1r^+^* B-ALL, AML	Upregulated	*CASC15* regulates expression of *SOX4* (B cell reg.) and YY1; overexpression opposes cellular proliferation and promotes myeloid bias in vivo; associated with a better prognosis	HIF1-alpha hypoxia sensitive elements within *CASC15* promoter	[96,97]
*ARIEL*	*TAL1*^+^ T-ALL	Upregulated	Enhancer RNA: recruits mediator proteins to the ARID5B enhancer, promotes enhancer-promoter interactions, activates ARID5B expression, thereby positively regulating the *TAL1*-induced transcriptional program and *MYC* oncogene	ARIEL transcription is activated by TAL1 complex	[92]
*LUNAR1*	*NOTCH*-regulated T-ALL	Downregulated	enhancer lncRNA: activates *IGF1R* expression, T cell proliferation	Regulated by *NOTCH1*	[88,90]
**Cytoplasmic Function: Protein Translation, mRNA Stability**					
*AS-RBM15*	AMKL	Downregulated	*AS-RBM15* promotes terminal differentiation by enhancing RBM15 translation in a 5′ cap-dependent manner. The overlapping region between *AS-RBM15 RNA* and 5′ UTR of *RBM15* mRNA function as enhancer of RBM15 protein synthesis	*AS-RBM15* transcription is activated by *RUNX1 and* repressed by *RUNX1-ETO*	[95]
*PU.1-AS*	AML	Upregulated	The simultaneous expression of both sense mRNA and anti-sense RNA (*PU.1-AS*) transcripts; *PU.1-AS* RNAs consist ~12–15% of *PU.1* mRNA level but are more stable than *PU.1* mRNA; *PU.1-AS* RNA forms complex with eIF4A and stalls PU.1 mRNA translation between initiation and elongation steps	Upstream regulatory element (URE) which physically interacts with both sense and anti-sense promoters; CBF fusions (*RUNX1-ETO* and *CBFβ-MYH11*) in AML	[102,103]
*UCA1*	AML, breast cancer, other types	Upregulated	hnRNP1 is a splicing factor that also promotes cap-independent translation through binding with IRES and recruiting ribosomes to p53 and p27 (Kip1) mRNAs. lncRNA *UCA1* binding with phosphorylated cytosolic form of hnRNP1 has anti-apoptotic effect in breast cancer. In leukemia, UCA1 sponges for miR-126, miR-125a, miR-16, and activates PI3K/AKT and JAK/STAT signaling	Regulated by CCAAT/enhancer-binding protein-alpha	[89,99,104,105]

**Table 2:** Long non-coding RNA in pediatric leukemia.

lncRNA Gene Name	Type of Cancer	Study Design	Expression in Cancer	Prognostic Significance or Function in Cancer	Ref
*SNHG1*	AML pediatric	newly diagnosed AML (*n* = 209), healthy controls(*n* = 67), BM, qRT-PCR	upregulated	shorter event-free and overall survival (*p* < 0.001)	[128]
*SOX6-1*	AML pediatric	de novo AML (*n* = 146), nonhematologic cancer controls(*n* = 73), BM, proliferation, qRT-PCR apoptosis CCK-8 and AV/PI assay	upregulated	poor-risk stratification, overall survival (*p* < 0.001),	[129]
*LINC00909*	AML pediatric	untreated AML (*n* = 93), healthy controls (*n* = 31), BM, RT-qPCR analysis, RNA-pull down; luciferase reporter assay; cell viability, migration	upregulated	sponge miR-625, activate WNT-signaling, poor prognosis, AML progression	[124]
*UCA1*	AML pediatric	UCA1 expression in AML (*n* = 27) before and after adriamycin (ADR)-based chemotherapy, cell lines, qRT-PCR, luciferase reporter assay, RIP	upregulated	chemoresistance, inhibits glycolysis through the microRNA-125a/hexokinase 2 pathway	[105]
*UCA1*	AML pediatric	untreated AML (*n* = 27), PB healthy donor controls, cell lines	upregulated	sustains AML proliferation similar to adult AML	[125]
*H19*	AML pediatric	gene expression profiles from 1361 childhood leukemia patients in 14 independent studies using available Affymetrix data	upregulated	*LIN28B and LIN28B*-driven *H19* expression present in aggressive subsets of pediatric leukemia	[120]
*ENST00000435695* *ENST00000415964*	AML pediatric	Arraystar Human IncRNA Array V3.0 in three AML vs. controls followed by qRT-PCR in AML BM (*n* = 22)	372 dysregulated IncRNAs (difference ≥ 10-fold)	*ENST00000435695* (most upregulated) *ENST00000415964* (most downregulated)	[133]
*lnc-THADA4-1 * *lnc-SUPT3H-1*	JMML pediatric	lncRNA landscapes in untreated JMML (*n* = 44, *n* = 19) and healthy BM donors, clinical and molecular characteristics, lncRNA-mRNA interaction network, LNA™ GapmeRs inhibition, cell viability	*lnc-THADA4-1 (highest)* *lnc-SUPT3H-1 (lowest)* lncRNA specific for granulocytic lineage– *lnc-ACSL1-1,lnc-BASP1-3*	Defined lncRNA associated with favorable and unfavorable prognosis, JMML lncRNA score: difference in the event-free survival from HSCT is significant, *p* < 0.0001	[131,132]
*TCL6* *CCDC26*	B-ALL pediatric	ETV6-RUNX1-positive (*n* = 24) versus ETV6-RUNX1-negative (*n* = 18) B-ALL, RNA seq, clustering analysis	*TCL6* *(* *highest* *)* ** *CCDC26* *(* *lowest)*	TCL6 levels may be associated with poor disease-free survival, even within ETV6-RUNX1-positive B-ALL (*p* < 0.05)	[130]
*CDKN2B-AS* *(ANRIL)*	B-ALL pediatric	genotype association study 217 B-ALL patients and 338 controls in CDKN2A/B (9p21.3) locus containing lnc-ANRIL	SNP	Six SNP inducing most strongly associated with B-ALL susceptibility rs2811712 located in the intron 1 on lnc-ANRIL	[21]
*BALR-2,* *(BALR-6,* *LIN00958)*	B-ALL pediatric	pediatric B-ALL *MLLr^+^, TEL-AML1*, *E2A-PBX1*, *BCR-ABL1* (*n* = 160)	Upregulated	Poor overall survival (*p* = 0.005)	[134]
*AWPPH*	T-ALL pediatric	de novo, untreated T-ALL (*n* = 32) healthy controls, BM, cell proliferation, apoptosis	upregulated	supports proliferation and inhibits apoptosis	[126]
*CDKN2B-AS1*	T-ALL pediatric	de novo untreated T-ALL (*n* = 21) and ADR-based therapies treated (*n* = 21), total T-ALL patients (*n* = 42), IP, RIP, Luc assay;	upregulated	ADR resistance, positive regulation of TRAF5 through miR-335-3p sponging	[127]
*INSR*	T-ALL pediatric	de novo, untreated T-ALL (*n* = 3) and healthy BM controls, anti-CD3 sorting, MNC RNAseq, lncRNA cellular localization	upregulated	lnc-INSR promotes tumor progression by promoting an immunosuppressive microenvironment in vivo	[122]
*NALT*	T-ALL pediatric	T-ALL (*n* = 20), BM, proliferation assay in vitro and in vivo PDX	upregulated	Co-expressed and supports NOTCH1 signaling, nuclear localization, novel cis-acting element regulating NOTCH1	[121]
